# A Dress Rehearsal for Assessment of Potency of Primordial Prevention for Reduction of Pandemic Stress Among Preadolescents

**DOI:** 10.7759/cureus.41173

**Published:** 2023-06-30

**Authors:** Nirupam N Sahu, Jaya Gawai

**Affiliations:** 1 Child Health Nursing, Smt. Radhikabai Meghe Memorial College of Nursing, Datta Meghe Institute of Medical Sciences, Wardha, IND; 2 Mental Health Nursing, Smt. Radhikabai Meghe Memorial College of Nursing, Datta Meghe Institute of Medical Sciences, Wardha, IND

**Keywords:** paying gratitude, self-character strength, self-compassion, values and beliefs, primordial prevention, preadolescents, stress, maharashtra

## Abstract

Background

Stress can have a significant impact on the mental health of both adults and children. Adolescents, in particular, are a vulnerable group, and the stress brought on by the pandemic may impact their prospects. This study serves as a preliminary assessment of the effectiveness of primordial prevention in mitigating pandemic-related stress among preadolescents.

Aim and objective

To perform a dress rehearsal to assess the effectiveness of primary prevention strategies in mitigating pandemic-related stress among preadolescents, the Perceived Stress Scale (PSS) in its English version was employed to measure the magnitude of stress experienced by the participants.

Material and methods

The study comprised a total of 100 preadolescent students who attended school in two distinct sections of Maharashtra, India. Each group consisted of 50 students initially, but after applying the inclusion and exclusion criteria, the number of students in each group was reduced to 35. The preadolescents in the experimental group underwent a pre-test using the PSS. Following this, they received training in a specific intervention that focused on five exercises related to positive psychology, namely, values and beliefs, self-compassion, knowing one's character strengths, and expressing gratitude. On the other hand, the control group did not receive any intervention. A post-test was conducted using the PSS checklist, and the scores were evaluated on the seventh day following the intervention.

Results

The preadolescents attending school had an average age of 12.5 years and were enrolled in the 7th and 8th grades. In the experimental group, most students identified as Hindus (30 individuals, accounting for 85.7% of the group), while a smaller portion identified as Buddhists (five individuals, making up 14.3%). In the control group, 25 students (71.4%) identified as Hindus, nine (25.7%) identified as Buddhists, and only one (2.9%) identified as Muslim. During the pre-test, 30 students (85.7%) from the experimental group and 28 (80%) from the control group exhibited moderate stress levels. Following the intervention, there was a significant improvement in the PSS scores from the pre-test to the seventh day. Specifically, in the experimental group, only eight individuals (22.9%) reported experiencing moderate stress; in the control group, the number was 28 (80%). The X2 value was calculated to be 22.88, with a level of significance set at p=0.0001.

Conclusion

Our dress rehearsal demonstrated the effectiveness of primordial prevention in mitigating pandemic-induced stress among preadolescent students attending schools in Maharashtra. The intervention employed five exercises rooted in positive psychology: emphasizing values and beliefs, cultivating self-compassion, fostering self-awareness of character strengths, and practicing gratitude. These interventions offer promising avenues for addressing stress among preadolescents in an educational setting. However, further research with larger sample sizes and longer follow-up periods is necessary to validate the effectiveness of these primordial preventive measures.

## Introduction

The global COVID-19 pandemic has had far-reaching consequences on the mental health and well-being of individuals across all age groups, including preadolescents. Preadolescence represents a critical developmental stage characterized by heightened vulnerability to stressors and the emergence of various psychological challenges [1,2]. During this transitional period between childhood and adolescence, preadolescents undergo significant physical, cognitive, and emotional changes, making them particularly susceptible to the negative effects of stress and adversity.

The prolonged exposure to pandemic-related stressors, such as social isolation, disruption of daily routines, and fear of infection, poses a substantial threat to the psychological well-being of preadolescents [3]. These stressors have the potential to exacerbate existing psychological distress and even contribute to the development of mental health problems among this vulnerable population. Recognizing the potential long-term implications, it becomes crucial to explore effective preventive strategies that can alleviate the adverse psychological impact of the pandemic on preadolescents.

One promising approach in this regard is primordial prevention, which aims to address risk factors at their roots and promote resilience proactively [2]. Primordial prevention strategies go beyond treating symptoms and instead focus on tackling the underlying determinants of mental health, fostering protective factors, and promoting well-being. By adopting a primordial prevention perspective, interventions can target modifiable factors related to preadolescents' environment, lifestyle, and social context, thereby reducing the impact of stress and enhancing their ability to cope effectively [4].

However, despite the potential benefits, there is currently limited research specifically examining the efficacy of primordial prevention interventions in the context of pandemic-related stress among preadolescents. Therefore, the objective of the present pilot study is to assess the potency of a primordial prevention program in reducing pandemic-related stress among preadolescents. The study aims to investigate the feasibility, acceptability, and initial efficacy of the intervention, shedding light on its potential effectiveness in promoting the psychological well-being of preadolescents during times of crisis.

## Materials and methods

Study setting and design

A randomized controlled noninferiority superiority trial was conducted in June 2022 at Salod Middle School in Wardha district, Maharashtra, India. The study received approval from the secondary school principal in Wardha district, Maharashtra, India.

Study population

A total of 70 preadolescents participated in the dress rehearsal, 35 in the control group and 35 in the experimental group. The study included students whose guardians provided informed consent. The preadolescents met the age criteria of 9-13 years and were enrolled in the selected institute for observation. Additionally, preadolescents who were absent during data collection were excluded.

Intervention

The preadolescents in the experimental group, who attended school, underwent a pre-test utilizing the Perceived Stress Scale (PSS). Subsequently, they received training in a targeted intervention centered around five exercises rooted in positive psychology. These exercises were designed to promote values and beliefs, self-compassion, understanding of personal character strengths, and the expression of gratitude. The intervention encompassed primordial prevention, as well as primary, secondary, and tertiary prevention approaches. In contrast, the control group did not receive any specific intervention but continued to receive primary, secondary, and tertiary prevention measures as per usual. The investigator assessed the intervention's primordial prevention aspect, while teachers conducted the assessments.

Data collection

The PSS was used to assess the participants' perceived stress levels. The checklist comprehensively encompasses all aspects of stress assessment among preadolescents, utilizing appropriate methods without cortisol levels (Appendices).

The stress levels of preadolescents were assessed by their class teachers using the PSS. Baseline data were collected initially to ascertain the preadolescents' stress levels. The experimental group received interventions categorized as primordial, primary, secondary, and tertiary prevention, while the control group received primary, secondary, and tertiary prevention.

The questionnaire inquiries about thoughts and feelings experienced in the past month. The respondents rate the frequency of these experiences on a five-point scale, ranging from 'Never' to 'Very often.' The responses are then scored as follows: 'Never' = 0, 'Almost never' = 1, 'Sometimes' = 2, 'Fairly often' = 3, and 'Very often' = 4. To calculate the total PSS score, the responses to the four positively worded items (items 4, 5, 7, and 8) are first reversed, meaning that the scores are inverted (i.e., 0 becomes 4, 1 becomes 3, 2 becomes 2, 3 becomes 1, and 4 becomes 0). The PSS score is then obtained by summing the scores across all items. Higher scores indicate higher levels of perceived stress. The scoring system for the PSS tool is divided into four levels as follows: low stress (0-13), moderate stress (14-26), and severe stress (27-40).

Statistical analysis

We analyzed demographic variables among preadolescents, including age, gender, father's education, father's occupation, income, mother's education, mother's occupation, socioeconomic status, and religion. Descriptive statistics were employed to determine the preadolescents' scores before and after the intervention, with chi-square tests, reliability assessments, and validity evaluations. We utilized SPSS version 27.0 and GraphPad Prism version 7.0 for our statistical analyses, and a significance level of p<0.05 was considered. The results are presented in tables and graphs, providing a comprehensive overview of the findings.

Reliability analysis

Using the parallel form reliability method, a value of 0.9711 was obtained, indicating that the tool demonstrates reliability and validity.

Ethical consideration

Each participant provided written informed consent following a thorough explanation of the study's concept and objectives. The study received approval from the Institutional Ethics Committee of Datta Meghe Institute of Medical Sciences (Deemed to be University). It was granted ethical approval (IEC/DEC-2020-21/9154). The study protocol was published in the Journal of Pharmaceutical Research International [5].

## Results

Table 1 presents the demographic characteristics of preadolescents in the experimental group (N=35) and control group (N=35). It includes information on age, gender, education of parents, occupation of parents, socio-economic status, and religion. Chi-square values and corresponding p-values were provided to assess any significant differences between the groups. The table indicates that both groups had preadolescents aged 12-13 years. Gender distribution showed no significant difference. The education, occupation, socio-economic status, and religion variables also did not exhibit significant differences between the groups.

**Table 1:** Distribution of preadolescents in the two groups as per their demographic nature

Demographic Variables	Experimental Group (N=35)	Control Group (N=35)	Chi-Square Value	P-Value
Age in years			-	-
10-11 yrs	0 (0%)	0 (0%)		
12-13 yrs	35 (100%)	35 (100%)		
Gender			0.52	0.46
Male	16( 45.7%)	13 (31.1%)		
Female	19 (54.3%)	22 (62.9%)		
Transgender	0 (0%)	0 (0%)		
Education of father			4.91	0.17
Illiterate	0 (0%)	0 (0%)		
Primary	2 (5.7%)	5 (14.3%)		
Secondary	8 (22.9%)	14 (40%)		
Graduation	12 (34.3%)	8 (22.9%)		
Others	13 (37.1%)	8 (22.9%)		
Education of mother			5.26	0.15
Illiterate	0 (0%)	0 (0%)		
Primary	2 (5.7%)	5 (14.3%)		
Secondary	10 (28.6%)	16 (45.7%)		
Graduation	14 (40%)	10 (28.6%)		
Others	9 (25.7%)	4 (11.4%)		
Occupation of father			5.95	0.20
Government	4 (11.4%)	6 (17.1%)		
Private	12 (34.3%)	9 (25.7%)		
Business	4 (11.4%)	7 (20%)		
Labor	1 (2.9%)	5 (14.3%)		
Others	14 (40%)	8 (22.9%)		
Occupation of mother			2.36	0.50
Government	3 (8.6%)	4 (11.4%)		
Private	4 (11.4%)	3 (8.6%)		
Business	2 (5.7%)	0 (0%)		
Labor	0 (0%)	0 (0%)		
Others	26 (74.3%)	28 (80%)		
Socio-economic status			4.26	0.37
Upper	1 (2.9%)	0 (0%)		
Upper Middle	13 (37.1%)	11 (31.4%)		
Lower Middle	11 (31.4%)	7 (20%)		
Upper Lower	4 (11.4%)	9 (25.7%)		
Lower	6 (17.1%)	8 (22.9%)		
Religion			2.59	0.27
Hindu	30 (85.7%)	25 (71.4%)		
Muslim	0 (0%)	1 (2.9%)		
Sikh	0 (0%)	0 (0%)		
Christian	0 (0%)	0 (0%)		
Others	5 (14.3%)	9 (25.7%)		

Table 2 shows the distribution of preadolescents' PSS scores in the experimental group and control group pre-test. The experimental group had four participants (11.4%) with low stress, 30 participants (85.7%) with moderate stress, and one participant (2.9%) with high stress. The control group had seven participants (20%) with low stress and 28 (80%) with moderate stress.

**Table 2:** Distribution of preadolescents according to their perceived stress scale score at pre-test in the two groups

Perceived Stress Scale Score	Experimental Group	Control Group	Χ2-value	P-Value
Low Stress	4 (11.4%)	7 (20%)	1.88	0.38
Moderate Stress	30 (85.7%)	28 (80%)		
High Stress	1 (2.9%)	0 (0%)		
Total	35 (100%)	35 (100%)		

Table 3 shows the distribution of preadolescents' PSS scores at the post-test in the experimental group. Out of the participants, 77.1% had a low-stress level, while 22.9% had a moderate stress level. No participants had a high-stress level.

**Table 3:** Distribution of preadolescents according to their perceived stress scale score at post-test in the experimental group

Perceived Stress Scale Score	Experimental Group	Control Group	Χ2-value	P-Value
Low Stress	27 (77.1%)	7 (20%)	22.88	0.0001
Moderate Stress	8 (22.9%)	28 (80%)		
High Stress	0 (0%)	0 (0%)		
Total	35 (100%)	35 (100%)		

## Discussion

The study results provide valuable insights into the distribution of preadolescents' perceived stress levels in the experimental and control groups at both the pre-test and post-test stages. At the pre-test stage, the experimental group had a higher percentage of participants with moderate stress (85.7%) than the control group (80%). However, the control group had a slightly higher percentage of participants with low stress (20%) than the experimental group (11.4%). These findings indicate that both groups experienced a considerable stress level, with the experimental group having a higher prevalence of moderate stress.

The distribution of perceived stress levels at the post-test stage in the experimental group showed significant improvement. Most participants (77.1%) reported low-stress levels, while only 22.9% reported moderate stress levels. Notably, no participants in the experimental group reported high-stress levels after the intervention. This indicates that the intervention program effectively reduced stress levels among preadolescents in the experimental group.

The findings of this study align with previous research that highlights the impact of intervention programs on reducing stress levels among adolescents. The study by Brody et al. demonstrated the long-term effects of a family-centered program in reducing conduct problems among African American youths [6]. Similarly, Brody et al. found that a family-centered program effectively deterred substance use, conduct problems, and depressive symptoms in black adolescents [7].

It is important to consider the potential influence of external factors on preadolescents' stress levels during the COVID-19 pandemic. Studies have highlighted the psychological impact of quarantine and the prevalence of psychiatric disorders among adolescents during the pandemic [8,9]. These factors might have contributed to the high levels of perceived stress observed in both the experimental and control groups at the pre-test stage.

The study's results demonstrate the effectiveness of the intervention program in reducing preadolescents' perceived stress levels. The experimental group showed a significant decrease in stress levels at the post-test stage, with most reporting low-stress levels. Conversely, the control group exhibited higher stress levels, indicating the need for targeted interventions. These findings support the importance of implementing intervention programs to alleviate stress among preadolescents, especially during challenging times, such as the COVID-19 pandemic. Further research and evaluations are necessary to determine such programs' long-term impact and explore additional factors that may influence stress levels in this population.

Despite significant efforts made by the government of India throughout the country, providing nationwide psychological interventions is nearly impossible due to the primary focus on locally meeting children's basic needs. A study revealed that insufficient access to basic supplies such as food, water, and clothing during quarantine resulted in frustration, anxiety, and anger that persisted even four to six months after release [10]. To mitigate the psychological impact, enhancing children's access to disease-related information through mediums such as comic books and videos and ensuring timely referrals to psychiatrists are crucial. Social media can also play a pivotal role by allowing quarantined individuals to keep their loved ones updated about their well-being. Consequently, having a functional mobile phone has transformed from a luxury to a necessity.

Limitations of the study

It is important to acknowledge the limitations of this study. The small sample size in both the experimental and control groups might limit the generalizability of the findings. Additionally, the study focused solely on perceived stress levels and did not consider other factors that could contribute to stress, such as socioeconomic status, family dynamics, or individual coping mechanisms. Future research could explore these factors to gain a more comprehensive understanding of preadolescent stress and effective intervention strategies.

## Conclusions

Primordial prevention strategies surpass symptom management and instead prioritize addressing the fundamental drivers of mental health, fostering protective factors, and promoting overall well-being. Embracing a primordial prevention perspective enables interventions to pinpoint modifiable factors about preadolescents' environment, lifestyle, and social context. Consequently, these interventions effectively diminish the impact of stress while bolstering preadolescents' resilience and capacity to cope adeptly.

## Appendices

**Table 4:** Perceived stress scale

Sr. No.	Questions	Never (0)	Almost Never (1)	Sometimes (2)	Fairly Often (3)	Very Often (4)
1	In the last month, how often have you been upset because of something that happened unexpectedly?					
2	In the last month, how often have you felt that you were unable to control the important things in your life?					
3	In the last month, how often have you felt nervous and stressed?					
4	In the last month, how often have you felt confident about your ability to handle your personal problems?					
5	In the last month, how often have you felt that things were going your way?					
6	In the last month, how often have you found that you could not cope with all the things that you had to do?					
7	In the last month, how often have you been able to control irritations in your life?					
8	In the last month, how often have you felt that you were on top of things?					
9	In the last month, how often have you been angered because of things that happened that were outside of your control?					
10	In the last month, how often have you felt difficulties were piling up so high that you could not overcome them?					
